# Editorial

**Published:** 1861-08

**Authors:** 


### Editorial.

Clinical Instruction and Medical Schools.—In a late number of the *American Medical Times*, the editor made some judicious remarks on the importance of a close connection between the Medical Colleges and Hospital Clinical instruction. In the article, he alludes to the Long-Island College Hospital, and the New York Medical College and Charity Hospital, as institutions presenting good examples in this respect. We wish to remind the editor of the *Times* that the two institutions just named, are not the first to set the example referred to. Fully six years before either of them had an existence, direct Hospital Clinical instruction, both in Practical Medicine and Surgery, was established as a part of the regular annual course in the Rush Medical College of this city.

The courses on Clinical Surgery were given by Professors Brainard, Herrick and Freer; those on Practical Medicine, by the senior editor of the EXAMINER. They were not mere collateral advantages, which the student might enjoy or not at his option, but the clinics were given *daily*, and every candidate for graduation was required to attend them as regularly as any part of the College course. In the establishment of the Medical Department of the Lind University in this city, two years since, Hospital Clinical instruction was made a still more prominent part of the curriculum of study. Regular Professorships of Clinical Medicine and Clinical Surgery were established, and *daily* clinical instruction at the bedside in the wards of the Mercy Hospital, was made a necessary part of the College course. And in addition to the daily clinic in the Mercy Hospital, the students of this University now have access to two clinics per week in the Marine Hospital, and one Surgical and one Medical clinic each week at the Chicago City Dispensary. It is true that the College and the Hospitals are not under the same roof, but they are only a few blocks apart, and the arrangement of all the clinical advantages is such as to constitute a prominent and harmonious part of the College course. Nor is the Clinical instruction in connection with the Lind University, limited to the ordinary winter term of the colleges, but it is continued to good classes throughout the whole year, except the month of August.

In no city in the United States, has Clinical instruction been made a more prominent or necessary part of the education of the medical student, than in Chicago, from the autumn of 1850 to the present time.

CHOLERA INFANTUM—SUMMER COMPLAINTS AND TEETHING.—At the present season of the year we can call the attention of our readers to nothing more important, than the following paragraphs taken from the introductory lecture of Dr. Jacobi, of New York, on the subject of "Dentition and its Derangements."

"You know that among the public at large, even among the educated portion of the community, *teething* is regarded as one of the two scape-goats of all diseases of infantile age.

Teething and worms are among mothers, acknowledged as the universal and all-powerful sources of disease."

"A mother will bring you her child, thin, emaciated, and anæmic, with sunken eyes and the wrinkled physiognomy of old age, and tell you she is well aware the poor thing is suffering from teething, and that, therefore, nothing can be done to alleviate its sufferings. *She will never be convinced that the child is dying from her own neglect;* that she has allowed a slight catarrh of the intestines, perhaps, to degenerate into incurable follicular ulceration. * * * * Teething is thus considered the most efficient cause of most of the terrible diseases, which prove fatal to thousands of the rising generation. *I can assure you that the readiness to attribute all the diseases of infantile life to teething, has destroyed more human beings than many of the wars described in history.* For, though parents are so much impressed with the belief of the dangers of teething, still they never think of attempting to save the lives of their children by counteracting the supposed life-endangering power of a normal process."

Again he says: "And it would be fortunate if the prejudice were confined to the public. But unfortunately, it still lingers in the medical profession, and it is for this reason that I have dwelt upon it thus lengthily. Nothing is more common than to hear doctors, young and old, in cases of infantile disease, diagnosticate *teething*, after mother and nurse had done so before, and nothing is more frequent than to be told that the death of a child was the consequence of dentition. Consider, for a moment, the absurdity of the conclusion, that a normal, physiological process is fatal to the existence of a living being. Who has ever ventured to assert that menstruation, pregnancy, or the climacteric years are the direct causes of death? *It is equally absurd to assert it of dentition*; and yet such statements are daily made by physicians."

The above extracts from one of the most eminent and experienced physicians of New York, accord exactly with our own sentiments. We regard the idea that the *natural* growth of the teeth produces disease just as absurd, as it would be to attribute disease to the growth of the hair, the toe-nails, or any other part of the body. And yet, as often as July and August comes, thousands of children, from three weeks to three years old, are attacked with various grades of gastric and intestinal irritation, which, in nine cases out of ten is attributed to "*teething.*" Every day our office is thronged with these little innocent beings, emaciated, eyes sunken, skin wrinkled, and vital properties exhausted, from a profuse, watery diarrhœa and occasional vomiting, of from one to three weeks' duration. When the mother is asked why she neglected the child so long, her prompt reply is, "O! the child is *teething.*" If we protest against the absurdity of the excuse, she often says "Doctor A. or B. told her the child was teething, and it would not do to stop the diarrhœa." And we are fully satisfied that more than *one hundred* children are sacrificed in this city alone, every summer, to the one single absurd error that because a child is *teething*, a diarrhœa need not be interfered with. Not only is the notion, that a strictly *normal* process like the growth of the teeth is the cause of disease, absurd, as represented by Dr. Jacobi; but, if it were true, instead of furnishing an excuse for neglecting the little sufferers, it should cause every one of common sense, whether mother, nurse, or physician, to watch them all the more carefully during the period of such growth.

### KEROSOLENE—Letter from Dr. Dickinson.

TO THE EDITORS OF THE CHICAGO MEDICAL EXAMINER.

Gentlemen:—At your request, I herewith communicate all the facts in my possession respecting a new anæsthetic, called Kerosolene,—some observations in relation to which, together with a sample, I had the pleasure of presenting to the Chicago Medical Society. Circumstances have prevented me from making many experiments with this interesting agent. I, however, presented a specimen of it to Dr. Gustave Weber, of Cleveland, and hoped to have his experience to embody in this communication: this must now be reserved till a future time. I have, at this time, only desired to bring it to the knowledge of the profession, by which its properties will be fully ascertained and appropriated accordingly.

WM. DICKINSON.

CHICAGO, ILL., July 20, 1861.

During the distillation of a peculiar kind of bituminous coal, in the manufacture of kerosene, other products are obtained, comprising a series most interesting in their physical properties, and not less so in their chemical reactions. These, almost endless in variety, have for many months engaged the attention of chemists, and especially of Dr. Hayes, State Assayer of Massachusetts.

In a casual conversation with Joshua Merrill, Esq., Superintendent of Downer's Kerosene Oil Works, Boston, Massachusetts, he brought to my notice this article—kerosolene—which, coming over early in the process, is condensed at a low temperature. This was, however, first discovered at similar works in New York city, formerly under the supervision of Mr. Atwood, chemist, and to it the name Kerosolene was given, not claiming for it chemical propriety, but, at the time, given for the lack of a better.

It has been ascertained to be a pure hydro-carbon, in which the carbon is slightly in excess, being highly inflammable, burning with a bright flame and with a little smoke. It is perfectly limpid and colorless as water, more volatile and of less specific gravity than ether; probably about 0.650, ether being 0.750. Almost tasteless, except for an instant when first applied to the tongue; it boils at about the temperature of the body; when favorably exposed, its evaporation is so rapid that water placed in a watch glass, in its midst, immediately freezes. Its odor bears a faint resemblance to kerosene and chloroform, but without the pungency of the former, and destitute of the irritating qualities of chloroform and ether upon the mucous membrane of the air passages. It forms a perfect solution with both, and also with water. It is a very powerful solvent, and for a long time has been used by the workmen for the removal of tar, grease, etc., from their clothes, no trace of its presence being left after evaporation.

The discovery of its anæsthetic qualities was the result of accident. Two men were directed to enter and cleanse a receiver, from which its contents, kerosene, had been drawn. Upon entering it from the top, they were at once immersed in an atmosphere of this vapor, its specific gravity being like that of carbonic acid, greater than that of air. Mr. Atwood says: "It is known that one of the men was subjected to this concentrated vapor for three-quarters of an hour, and the other for a shorter period. When removed, both being completely narcotized, and permitted to respire pure air, they speedily recovered consciousness and the exercise of all their faculties, without suffering at the time or subsequently, the least inconvenience from the involuntary anæsthesia, of which they had been the first subjects." A similar accident, under like circumstances, occurred under the observation of Mr. Merrill. While recovering after being exposed to the free access of air he exclaimed, "What heavenly music! what delicious music!" These all unite in their testimony that no headache, vertigo, nausea or other disagreeable sensation was experienced, but were all able in a few moments to resume their respective duties, as though they had not made, unwittingly, perhaps a very valuable contribution to science.

Flies subjected to the vapor are quickly narcotized, but generally soon recover. In some instances after apparent recovery, they seem to die from its effects. So far as its effects upon mice have been observed, it has produced death, either immediately or at variable times after apparent recovery. This effect must not be arrayed specially against the safety of kerosolene, for both ether and chloroform often produce death upon the lower animals; and from the effects of the latter, patients have seemed to die, even after complete and safe recovery from the anæsthesia produced by it.

Here now, without question, was a new anæsthetic,—its peculiar properties and powers, undeveloped and unknown, a few inspirations of its vapor are sufficient to convince one that its power exceeds that of ether, and besides that it is more agreeable and much less irritating to the respiratory organs, I thought, if brought to the knowledge of the profession, and made the subject of judicious experiment, it might be found to possess more than mere kindred properties to ether and chloroform; perhaps to combine the power of the latter with the safety of the former. Accordingly, Mr. M. and self took a quantity of it to Dr. Cabot, one of the surgeons of Massachusetts General Hospital, and at the suggestion of Dr. Bowditch and assent of Mr. M., I introduced it to the Society for Medical Improvement, on the evening of 16th ult. Upon being presented by Dr. Bowditch, it elicited much interest, and was referred to the surgeons of the hospital and Dr. Bacon, professor of chemistry, for their investigation, experiment and report.

Dr. Bigelow, also one of the surgeons referred to, informed me, he was so firmly convinced of its innocency, that without further experiment, on the following day he caused it to be administered to himself by Dr. Hodges, with complete success in producing anæsthesia. The sensations he describes in the Boston *Medical and Surgical Journal** of the day following, as wholly agreeable, "leaving neither headache nor nausea, nor bad taste."

At the request of Dr. Andrews, of this city, I administered kerosolene to a lad about seven years, whom he was desirous of examining for suspected calculus. About one drachm of the liquid was applied to the surface of a folded handkerchief; a large admixture of pure air was thus allowed. Great caution was practiced in its administration, and the consequent phenomena carefully scrutinized. The inhalation was kindly borne, the vapor exciting neither apprehended suffocation nor even cough; but as consciousness declined, a general muscular rigor supervened, during which the respiration was suspended, and the face flushed, while the heart's action was increased both in frequency and force. A moment's removal of the handkerchief was followed by relaxation of the muscles. The inhalation was twice repeated with similar results. The pulse, at no instant, either flagged or intermitted; urine was voided involuntarily.

In the present state of our knowledge, ten days not yet having elapsed since its first administration to the human subject in

---

* *Vide* page 408—EXAMINER, for Dr. Bigelow's article.—R.

the person of Dr. Bigelow, we deemed it hazardous to persist in the use of kerosolene; it was therefore suspended, and the anæsthesia concluded by a mixture of ether and chloroform, of which the latter bore but a small proportion. The quantity of kerosolene employed, did not exceed one-half an ounce. Further experience may demonstrate that the rigor is but an innocent index of commencing anæsthesia, and has no value as a sign of danger; that it consequently can, with entire safety be disregarded; and also, that relaxation would follow upon a more complete state of anæsthesia, in the same manner as muscular action, or resistance during the administration of ether or chloroform, is an unequivocal indication to persist for the purpose of overcoming such action,—or it may be an indication to withhold its administration for a moment—the rigor having the same relation to the inhalation of kerosolene as suspended respiration to that of the other anæsthetics. Should it be ascertained that kerosolene, in its present state of manufacture, possesses positively noxious qualities, a process of purification may be adopted, which will entirely divest it of such properties.

Should experience award to it the power and safety alluded to above, its cheapness, in addition to other considerations, will strongly conspire to render it the favorite anæsthetic, since I have the assurance of Mr. Merrill that it can be furnished at one dollar a gallon.

### THE NEW SPLINTS FOR HIP DISEASE.

TO THE EDITORS OF THE EXAMINER.

Gentlemen:—In the editorial department of the *Medical and Surgical Reporter*, Dr. O. C. Gibbs, of Frewsbury, N. Y., gives a notice of the three splints devised by Dr. Davis, Dr. Sayre, and myself, for the better treatment of morbus coxarius. In the course of his highly complimentary remarks, he inquires very naturally as follows:

"We do not see how he (Dr. Andrews) secures immobility of the hip joint. Will the Professor tell us if he regards this as non-essential."

In reply to this I would state that immobility of the joint *after excision* is not desired, because the object sought is a ligamentous union of the cut extremity of the femur to the ilium. In cases not requiring excision, immobility would be desired, were it attainable without the confinement of the patient, but this is impossible. Neither Davis' nor Sayre's splints attain it, nor can any other which allows the patient to walk about freely. Slight motion of the joint when it is firmly extended is of but little consequence. When no splint is used, the patient at each step brings the whole weight of the body to press and grind on the inflamed head of the femur, hence the pain and the real mischief which result from attempts at walking in such instances, but if a good splint is applied, the head of the bone is kept drawn a little off from the acetabular surface, and the weight of the body does not rest upon it at all. Slight motion in such circumstances does little or no harm, as is evident from the fact that the patients no longer suffer from it. The great merit in the splints of Drs. Davis and Sayre is not in aiming at immobility, but in taking off effectually all pressure from the joint. Probably my splint allows a little more freedom to the limb than theirs, but I have yet seen no injury from it.

I think that my splint presents one advantage over the others, dependent upon the fact that it transmits the weight of the person directly to the ground. In Sayre's method the weight is transmitted from the lower end of the instrument, through adhesive straps, to the skin of the leg or thigh, and thence through the foot to the ground. Now, as the skin is elastic, it yields a little at each step, and relaxes the extension, and hence, in some cases it is difficult to tell whether a part of the weight is not still borne by the inflamed head of the femur. In my splint I place the patient in a standing posture, and extend the instrument until the heel, in stepping, no longer presses the bottom of the shoe. I fear that Dr. Sayre's recent improvement, in which he uses a shorter splint, and applies his adhesive straps to the integument of the thigh, will be still more liable to the objection above mentioned from the elasticity and mobility of the skin.

Dr. Gibbs complains that my splint, being of Western origin, is overlooked at the East; but if there is any one to blame for that, it is probably myself. Having been occupied with my practice, I have neglected to take any measures to introduce it to the notice of the profession of that region. I have been called upon to see a great number of cases of hip disease, and merely devised this instrument for use among my own patients. Afterwards, I published an account of it in the EXAMINER of Chicago, but as I have taken no pains to bring it into notice further East, there are very few there who know of its existence.

Yours truly,

E. ANDREWS.

CHICAGO, July 24, 1861.

---

SURGEONS FOR THE ILLINOIS VOLUNTEERS.—The Board of Medical Examiners for this State, Prof. H. A. JOHNSON, M. D., President, resumed their sessions in this city on the 6th inst., at the rooms of the Indian Agency, in the Custom House building. Up to this date they have passed twenty-three *Surgeons*, as follows: Drs. W. Wagner, Geo. Coatsworth, H. Wardner, T. W. Winer, A. W. Heise, Jas. Bringhurst, C. Goodbrake, L. D. Kellogg, Wm. J. McKim, Henry Parker, Charles Storch, W. R. Burke, R. L. Metcalf, S. T. Trowbridge, W. M. Gray, R. G. Bogue, G. S. Lucas, L. Watson, S. C. Blake, S. M. Hamilton, S. W. Everett, Jas Roberts, and J W. Tuttle.

And twenty-five *Assistant Surgeons*, as follows: Drs. T. Babb, John Fetzer, F. K. Bailey, A. Blake, Carl Muntz. J. S. Hunt, S. C. Plummer, George H. Dewey, P. H. Bailhache, Sanford Bell, Chas. Davis, O. G. Hunt, D. Stahl, Jas. Hamilton, H. A. Buck, Edwin Gaylord, John B. Ensey, Jas. Farnum, E. Gulich, Benj. F. Stevenson, O. B. Ormsby, C. B. Tompkins, John M. Phipps, Wm. F. Cady, Theodore Blut hardt.

The Board still have some thirty-five or forty candidates on the list, and applications are made every day in excess of the examinations, so that their session bids fair to be indefinitely prolonged.

